# Cutting the Stone: Health Defined in the Era of Value-based Care

**DOI:** 10.7759/cureus.1023

**Published:** 2017-02-10

**Authors:** Ger Rijkers

**Affiliations:** 1 Science Department, University College Roosevelt

**Keywords:** immune system, vaccination, global health

## Abstract

The immune system contributes to the maintenance of health by preventing and limiting the clinical consequences of infections by pathogenic microorganisms. During the evolution of Homo sapiens, those with the fittest immune system survived. The immune system of Homo sapiens was further improved and adapted by admixture with Neanderthal genes. Nowadays, the human immune system provides adequate protection against the majority of infections. For some 20 infectious diseases, the immune system needs to be improved by vaccination. Vaccination is the number one value-based healthcare intervention and has resulted in global eradication of smallpox. Eradication of poliomyelitis and measles is within reach. A continuous effort will be required for recently emerged pathogens, such as Ebola and HIV, as well as the most difficult - malaria and tuberculosis.

## Introduction and background

**Figure 1:**
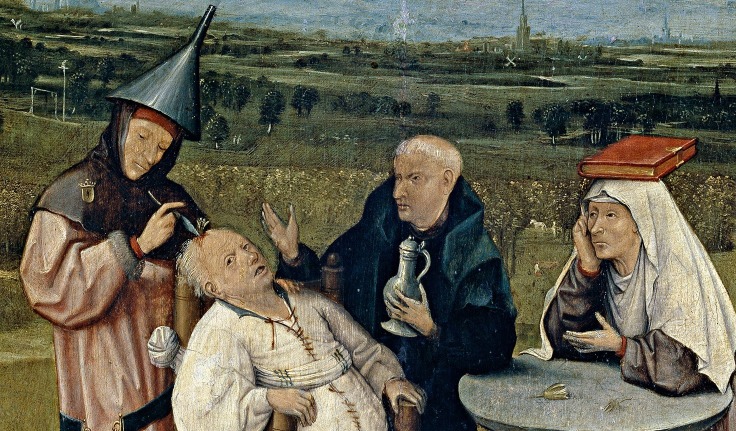
Cutting the Stone, Jheronimus Bosch, 1494. Museo Prado, Madrid.

The vast majority of medical professionals devote all their knowledge, energy, and compassion to treat patients who are not healthy. In a value-based care system, those treatments are evidence-based and there is no room for quackery. Jheronimus Bosch, in his 1494 painting entitled "Cutting the Stone", depicts an open-air event that clearly is a surgical intervention: trepanation (Figure 1). The patient is tied to his chair because the discovery of ether anesthesia would take more than 350 years. The text on the painting reads "Master, cut the stone out, my name is Lubbert Das." In those days, cutting a stone from someone’s head (or, like a magician, pretend to cut a stone from someone’s head) was considered to be a cure for stupidity and madness [1]. The alternative title of this painting is "The cure of folly". This would suggest that such an intervention actually is an effective treatment and would restore mental health. Bosch clearly depicts the procedure as not so value-based: the healthcare professional wears an upside down funnel, the spectators (a priest and a nun) are obviously bored, and the nun is balancing a locked book on her head. Above all, the object removed from the forehead of Lubbert is not even a stone but a tulip bulb. The Dutch word for tulip bulb (tulpenbol) in those days also meant fool or knucklehead.

Lubbert Das probably never lived. It was an imaginary figure, representing the simple-minded soul, an easy victim of charlatans who pretended to be a medical professional. Five hundred years ago, medical knowledge was limited and restricted to those who could read. Medical knowledge has exploded, and although we live in an information overload age, we should not underestimate the number of Lubbert Dasses of today who seek value-based health care.

## Review

So what do you care for in health care? According to the WHO definition, health is a state of complete physical, mental, and social well-being and not merely the absence of disease or infirmity [2]. This definition has not been amended or changed since 1948. "How do you do?” is probably the most common opening sentence in daily conversation. It is an all-inclusive inquiry about someone’s health, not just physical health. In most cases, "How do you do?" is answered in a single word: fine, great, so-so. When the person who is greeted struggles with his or her health, the answer can be more elaborate and then inevitably includes the dimension of time: deterioration (You can’t always get what you want) or improvement (It’s getting better all the time) of health. Most of the time, it will also include a prediction of future health.  

At the beginning of humankind, health was not a given thing but a constant struggle for life and only the fittest survived -- fittest not in the sense of physical fitness but best fitted to the circumstances. The ability to adapt, therefore, is crucial for survival and equally crucial for health. In this light, Machteld Huber and colleagues have taken on the brave task to redefine health [3-4]. According to their proposal, health is not a fixed state but a dynamic process, the ability to adapt and have self-control, in the context of the social, physical, and emotional challenges of life. Let us take this concept from the beginning, approximately 200,000 years ago, when Homo erectus evolved into Homo sapiens. For the first 100,000 years of their existence, Homo sapiens lived in Africa, but then, for unknown reasons, a number of them (probably just a small number) migrated out of Africa to Europe and Asia [5-6]. When Homo sapiens arrived in middle and northern Europe, Neanderthals already inhabited that ecological niche; in fact, they had already lived there for more than 200,000 years. Today, about 2% of our human DNA is Neanderthal DNA [7]. Not just in people with red hair, a supraorbital torus, or a pronounced jaw, but also in each of us, 2% of the DNA is derived from Neanderthals. Why is that? Why did Neanderthal DNA survive because the Neanderthals themselves did not survive; they are extinct. Quite a lot is known about Neanderthals, where they lived and how they lived, but not what happened to them after the arrival of Homo sapiens. At least three different scenarios can be proposed for their disappearance: one is that Homo sapiens killed all Neanderthals, maybe even ate them. A second scenario is that they may have been expelled to remote areas where they could not survive. The third scenario, maybe the most likely, is that Homo sapiens and Neanderthals did meet and mate, a sort of 2.0 version of kiss and ride. Thus, Neanderthal genes would be mixed with Homo sapiens DNA, which ultimately would have made the Neanderthals invisible. Actually, Neanderthals and Homo sapiens lived together for about 25,000 years, so that should be sufficient for an elaborate exchange of genes. Then why not end up with 50%, 25%, or 10% Neanderthal DNA? That may have been the result of subsequent selection pressure and survival of the fittest. Maybe the babies and children with the most Homo sapiens genes would be healthier and had a better chance to survive? If that were the case, then why, 50,000 years later, did we not end up with zero percent Neanderthal DNA? What is so important about the 2%? What is that 2% coding for?   

The answer to this question is found in a group of genes called human leukocyte antigens (HLA). These genes are extremely polymorphic, and this is important for the functionality of the immune system. This importance can be illustrated by kidney transplantation, a life-saving intervention for patients with end-stage kidney failure. After transplantation of a kidney, the immune system of the recipient will recognize the donor kidney as foreign and reject it. Only when the immune system of the recipient is suppressed can the kidney be accepted. The immune system recognizes the kidney as foreign because of differences in HLA molecules between donor and acceptor; nevertheless, when you think about it, that cannot have been the evolutionary driving force for the polymorphism of HLA molecules. Neanderthals did not practice kidney transplantation and neither did Homo sapiens, for that matter. Then what would be the function of HLA and its polymorphism? The biological function of an HLA molecule is to present fragments of microorganisms to the cells of the immune system so that it can be recognized. The better the presentation by HLA, the better the immune response and the better protection against an infection with that particular microorganism. However, there is not a single HLA molecule that can present the fragments of all microorganisms the best. Thus, this mechanism comes with a price: when you have inherited HLA molecules, which are very good at presenting influenza virus proteins, those molecules could be poor presenters of yellow fever virus proteins. In that case, your immunity against influenza would be excellent, but you would be more susceptible to yellow fever. That would constitute an individual risk: benefit ratio and that ratio will contribute to the changes of survival of that individual. At the population level, it is a different situation. For the survival of the population, it is important that an epidemic (for instance, with the black plague) does not wipe out the total population but that at least some individuals will survive. For the population, a high level of HLA polymorphisms, therefore, contributes to survival. Now back to the Neanderthals. It was found that among the HLA genes, the contribution of Neanderthal DNA is much higher than 2%, in some gene regions even as much as 20% [8-9]. The HLA genes and the immune system of the Neanderthals apparently were better adapted to the European and Asian conditions than that of the Homo sapiens. Neanderthal HLA genes were functionally beneficial for survival. The process of natural selection subsequently increased their frequency. In biology, this is called adaptive introgression. We should be thankful to our great-great-great-grandfather (or mother, for that matter) for having had sex with a Neanderthal.

Thus, the struggle for life and survival of the fittest could, or even should, be interpreted as survival of those with the fittest immune system. Over the ages, the fitness of the immune system has been tested repeatedly by epidemics of the black plague, smallpox, and many others. Thus, it could be expected that the current human population, the survivors, now have the almost perfect immune system, which should keep us healthy all of our life.

The ability to survive not only depends on the fitness of the immune system but also on the strength of the opponents, the microbiological opponents. Thus, how fit is our modern day immune system actually and does it function well enough to keep us healthy for all of our life? Apart from protection against infection, the immune system offers protection against malignancies: the immune system recognizes transformed cells as being foreign and attacks and kills those cells. Also from this perspective, the immune system contributes to maintaining health.

Nevertheless, let us start with the function of the immune system at the beginning of life, not 200,000 years ago, but now in 2017 when on our planet approximately 350,000 babies will be born on a given day. Each of those babies is born in a world surrounded by microorganisms. Fetal development itself takes place in the protected sterile environment of the womb. During fetal development, all organ systems are formed, including the immune system. Directly after birth, the human newborn and its immune system are challenged by being exposed to the great variety of potentially pathogenic microorganisms. The first critical period in surviving life, therefore, is during childhood. At the end of 1564, 60% of all babies born that year in Stratford upon Avon had died from the plague: Hic incipit pestis [10]. This made Bill Bryson, one of the many Shakespeare biographers, state that the biggest achievement of Shakespeare was not that he wrote Hamlet or anyone of his 154 sonnets but having survived his first year of life [11].

Every infection, being it with Yersinia pestis or another bacterium or virus, leads to a battle between the microorganism and the immune system. If the immune system wins, the microorganism loses, and you will survive. Surviving a childhood disease subsequently provides you with lifelong immunity. This is because the immune system of the newborn already is fully developed and can respond to whatever microorganism that challenges it. For most pathogens, the immune response is powerful enough; remember that we are survivors, and we have a Neanderthal-strong immune system. For a handful (well, maybe two or three handfuls) of microorganisms, the immune system is not powerful enough. These microorganisms include Clostridium tetani, Corynebacterium diphtheriae, and Bordetella pertussis. In order to survive an infection with these microorganisms, the immune system needs to be improved by vaccination. How devastating these infectious diseases can be, we can learn from history or by visiting countries where childhood vaccination has not yet been implemented and children die from these diseases.

Maintaining health by harnessing the immune system (vaccination) has been the most cost-effective intervention in medicine, certainly in a value-based healthcare system. Its direct effect is to prevent disease in the vaccinated child (or adult); its indirect effect is to protect the community, including those who otherwise would be vulnerable. Mass vaccination has led to the global eradication of smallpox [12], and poliomyelitis and measles hopefully will follow in the foreseeable future [13-14]. The development of an efficient Ebola vaccine shows that the international biomedical scientific community is capable of responding fast and adequately [15-16]. 

## Conclusions

Modern medicine is in an active transition from combatting disease to promoting health. Prevention of disease by vaccination thus contributes to the maintenance of health. Vaccination, therefore, improves the health of the individual who is vaccinated as well as the health status of the population at large. The path to health maintenance in the sense of prevention of infectious diseases is, therefore, clear. For a number of major diseases, vaccines are available and have been implemented. Clearly, the road ahead still has many challenges for the future, including effective vaccines for tuberculosis, malaria, and HIV as well as parasitic diseases. Hopefully, they will be able to provide value-based health for Lubbert Das as well as everyone else. 
